# Complications of thoracoscopic talc insufflation for the treatment of malignant pleural effusions: a meta-analysis

**DOI:** 10.1186/s13019-021-01475-1

**Published:** 2021-05-04

**Authors:** Wen Zhang, Yun-long Zhao, Shao-jun Li, Ying-nan Zhao, Nan-nan Guo, Bo Liu

**Affiliations:** Department of Chest Surgery, The Fourth Medical Center of PLA General Hospital, No.51, Fucheng Road, Haidian District, Beijing, 100048 China

**Keywords:** Talc insufflation, Malignant pleural effusions, Pleurodesis, Thoracoscopy

## Abstract

**Background:**

Talc pleurodesis is an effective treatment for malignant pleural effusions (MPEs). This study was designed to estimate complication rates of thoracoscopic talc insufflation.

**Methods:**

Literature search was conducted in electronic databases and studies were selected if they reported complication rates of thoracoscopic talc insufflation in cancer patients with MPEs. Meta-analyses of proportions were performed to obtain incidence rates of complications.

**Results:**

Twenty-six studies (4482 patients; age 62.9 years [95% confidence interval (CI): 61.5, 64.4]; 50% [95% CI: 43, 58] females) were included. Intraoperative, perioperative, 30-day, and 90-day mortality rates were 0% [95% CI: 0, 1], 2% [95% CI: 0, 4], 7% [95% CI: 3, 13] and 21% [95% CI: 5, 43] respectively. Incidence rates [95% CI] of various complications were: pain (20% [1, 2]), fever (14% [3, 4]), dyspnea (13% [5, 6]), pneumothorax (6% [7, 8]) pneumonia (4% [0, 12]), emphysema (3% [3, 7]), prolonged air leakage (3% [0, 7]), prolonged drainage (3% [9, 10]), thromboembolism (3% [9, 11]), lung injury (2% [7, 12]), respiratory insufficiency (2% [0, 5]), re-expansion pulmonary edema (1% [0, 3]), empyema (1% [0, 2]), respiratory failure (0% [0, 1]), and acute respiratory distress syndrome (ARDS; 0% [0, 1].

**Conclusions:**

Whereas pain and fever were the most frequent complications of thoracoscopic talc insufflation, the incidence of ARDS was low. Pneumothorax, pneumonia, emphysema, prolonged air leakage, pulmonary embolism, arrythmia, re-expansion pulmonary edema, and empyema are important complications of thoracoscopic talc insufflation.

**Supplementary Information:**

The online version contains supplementary material available at 10.1186/s13019-021-01475-1.

## Background

Malignant pleural effusion (MPE) is a fluid containing serous proteins, and lymphatic/myeloid cells which along with the cancerous cells accumulates in the pleural cavity during the advanced and terminal stages of cancer. This happens when normal fluid dynamics are disturbed due to the infiltration of tumor into thoracic lymph nodes and pleura [1]. MPEs develop more commonly in lung carcinoma, breast carcinoma, and lymphoma. Up to 15% of lung cancer patients and 11% of breast cancer patients develop MPEs during disease course [2]. Global incidence of MPEs is unknown. In the USA, annual incidence of MPEs is estimated to be between 150,000 and 250,000 cases [2–4].

Although, MPEs can be asymptomatic but most of the cases are symptomatic. Common symptoms are dyspnea, cough, and chest pain. MPEs are associated with 3–12 months shorter survival depending on the type of pathology. In lung cancer patients with MPEs, the survival is low (3–6 months), but survival is better (5–24 months) in breast cancer patients [5, 6]. Patients with MPEs have a diminished quality of life due to symptoms such as the shortness of breath [7]. Because patients with MPEs usually have advanced cancer therefore treatments are focused on palliative relief of symptoms. Although, therapeutic thoracentesis can provide relief, the prevention of fluid accumulation in the pleural cavity requires more invasive procedures. Generally, this is accomplished either by the implantation of an indwelling pleural catheter or by performing pleurodesis in which an inflammation-causing material such as talc, tetracycline derivatives, silver nitrate, povidone, or antineoplastic is administered into the pleural space to either through a chest tube or by thoracoscopy [7, 8].

Pleurodesis is an invasive and hospitalization requiring intervention for the resolution of MPEs. It can be performed as a thoracoscopic procedure or as video-assisted thoracic surgery [5]. Preoperative indicators including the high number of white blood cells, elevated alanine transaminase levels, body mass index < 18, hypoxemia and hypoalbuminemia are associated with worse survival after talc pleurodesis [9]. Prior chemotherapy, radiotherapy and poor performance status are also associated with poor prognosis [10, 11]. Pleurodesis with thoracoscopic talc insufflation (also called talc poudrage) is found to be more effective than other contemporary treatments for MPEs [12]. A network meta-analysis that evaluated pleurodesis failure rates found talc insufflation to be the best method of MPE treatment. Other pleurodesis agents which followed talc in ranking were talc slurry, mepacrine, iodine, bleomycin and doxycyline [13]. However, there is no comprehensive review of the complications of thoracoscopic talc insufflation in literature. The aim of the present study was to estimate the complication rates of thoracoscopic talc insufflation by performing meta-analyses of the incidence rates reported by the individual studies.

## Methods

### Eligibility criteria

Studies were included if they investigated the effectiveness of pleurodesis with thoracoscopic talc insufflation for the treatment of MPEs of cancer patients in a prospective or retrospective design and reported the complications of the procedure. Studies were however excluded if they a) reported the complications of talc slurry; b) involved more than one types of procedures and reported the outcomes without differentiation of treatments; c) reported the outcomes of talc insufflation in patients with pneumothorax; and d) case reports.

### Literature search

The literature search was conducted in electronic databases (Ebsco, Google Scholar, PubMed, Science Direct, and Wiley) using relevant key terms in logical combinations. These included malignant pleural effusion/s, thoracoscopy, thoracoscopic, pleurodesis, talc, insufflation, poudrage, intraoperative, postoperative, recurrence, complication/s, adverse effects / events, survival, mortality, cancer, and malignancy. The literature search encompassed research articles published in English before December 2020. Bibliographic lists of selected research and review articles were also screened to strengthen the literature search.

### Data analyses

Data pertaining to patient demographics, study design, follow-up, talc dose, presenting symptoms and performance status of patients, pleurodesis features, hospital stay, success and recurrence rates, survival, and adverse events / complications were extracted from the research articles of respective studies. Quality assessment of the included studies was performed with New Castle-Ottawa Scale for the Assessment of the Quality of Observational Studies or with Cochrane Risk of Bias Assessment Tool for Randomized Controlled Trials.

For each complication of thoracoscopic talc insufflation, a meta-analysis of proportions was performed to achieve the overall estimate where 95% confidence intervals of the estimate were calculated with the use of score statistics and the exact binomial method. In these meta-analyses, within-study variability was estimated from the binomial distribution. Freeman-Tukey double arcsine transformation was incorporated for variance stabilization in each meta-analysis [14]. Meta-analyses of proportions were also performed to achieve the estimates of success and recurrence rates. Survival rates reported by the individual studies were pooled under random-effects model using DerSimonian and Laird method for pooled estimates. All analyses were performed with Stata software (Stata Corporation, Texas, USA).

## Results

**Fig. 1:**
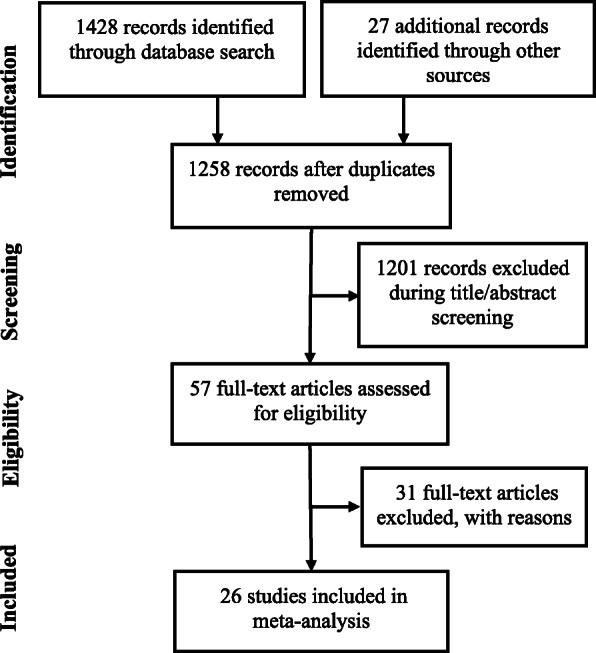
A flowchart of study screening and selection process

Twenty-six studies [15–40] were included (Fig. 1). Of these, 5 were randomized controlled trials, 4 prospective studies and 17 were retrospective studies. Overall, these studies reported the outcomes of 4482 patients. Average age of these patients was 62.9 years [95% confidence interval (CI): 61.5, 64.4]. The percentage of females was 50% [95% CI: 43, 58]. Important characteristics of the included studies are presented in Table S1. In general, the quality of the included studies was moderate to high (Table S2a&b).

Average dose of talc used in these studies was 4.57 g [95% CI: 4.02, 5.11]. On average, thoracoscopic pleurodesis was completed in 35 min [27, 41], chest tube drainage was completed in 4.5 days [95% CI: 3.9, 5.0], and patients required a hospital stay of 6.2 days [95% CI: 5.6, 6.9]. Intraoperative, perioperative, and 30-day, and 90-day mortality rates were 0% [95% CI: 0, 1], 2% [95% CI: 0, 4], 7% [95% CI: 3, 13], and 21% [95% CI: 5, 43] respectively (Figure S1). Pleurodesis success rate was 86% [95% CI: 82, 91]. Recurrence rate was 15% [95% CI: 7, 26] in a median follow-up duration of approximately 18.3 months [95% CI: 10.5, 26.0]. Median survival after talc insufflation was 7.8 months [95% CI: 6.4, 9.2].

**Fig. 2:**
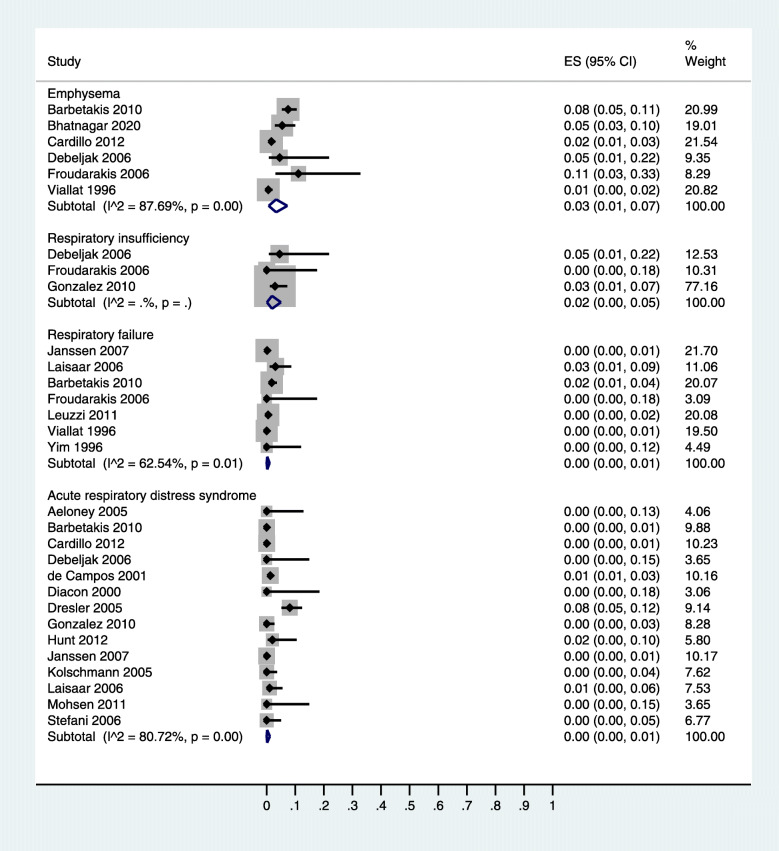
A forest graph showing the incidence rates of dyspnea, emphysema, respiratory insufficiency, respiratory failure, and acute respiratory distress syndrome after thoracoscopic talc insufflation

**Fig. 3:**
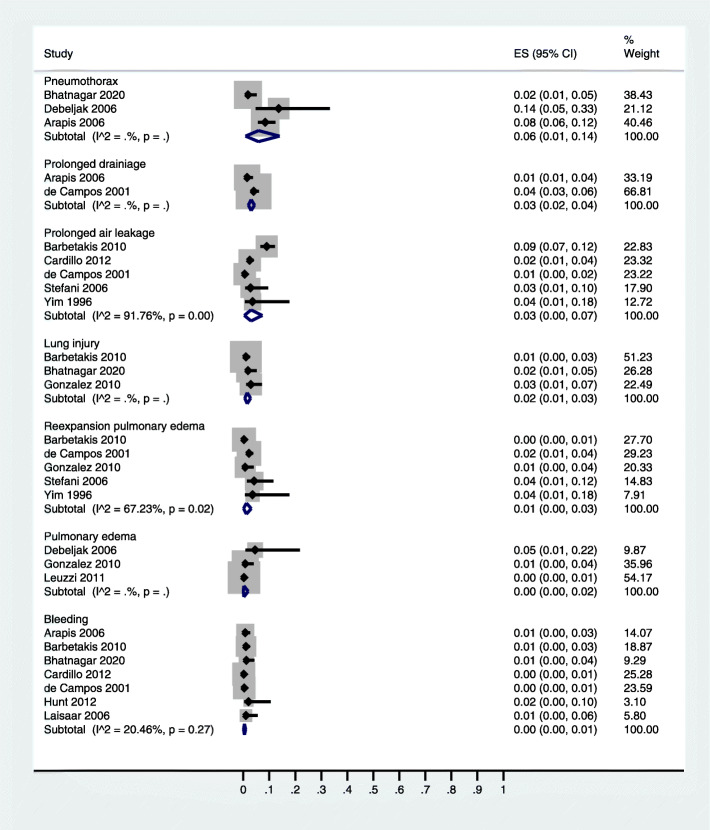
A forest graph showing the complication rates of persistence of fluid, pneumothorax, prolonged drainage, prolonged air leakage, lung injury, re-expansion pulmonary edema, pulmonary edema, and bleeding after thoracoscopic talc insufflation

Pain and fever were the most prevalent complications of thoracoscopic talc insufflation (20% [95% CI: 8, 37] and 14% [95% CI: 7, 23] respectively) (Table 1). Among the respiratory complications, emphysema (3% [95% CI: 1, 7]) was followed by the respiratory insufficiency (2% [95% CI: 0, 5]), respiratory failure (0% [95% CI: 0, 1]), and acute respiratory distress syndrome (ARDS; 0% [95% CI: 0, 1] (Table 1; Fig. 2). Among the pleural complications, pneumothorax (6% [95% CI: 1, 14]) was followed by the prolonged drainage (3% [95% CI: 2, 4]), prolonged air leakage (3% [95% CI: 0, 7]), lung injury (2% [95% CI: 1, 3]), re-expansion pulmonary edema (1% [95% CI: 0, 3]), and pulmonary edema (0% [95% CI: 0, 2]) (Table 1; Fig. 3).

**Table 1:** Complication rates of talc insufflation for MPEs

Complication	Number of studies	Number of cases	Total patients	Incidence [95% CI]	***p***-value
Pain	8	165	1378	20 [8, 37]	< 0.001
Fever	11	190	1574	14 [7, 23]	< 0.001
Pneumothorax	3	29	461	6 [1, 14]	< 0.001
Pneumonia	6	52	1058	4 [0, 12]	0.02
Emphysema	6	54	1577	3 [1, 7]	< 0.001
Prolonged air leakage	5	57	1661	3 [0, 7]	0.01
Prolonged drainage	2	26	823	3 [2, 4]	< 0.001
Thromboembolism	2	17	496	3 [2, 5]	< 0.001
Nausea/vomiting	3	5	234	2 [0, 5]	0.04
Respiratory insufficiency	3	5	178	2 [0, 5]	0.02
Hypotension	2	5	188	2 [0, 5]	0.01
Lung injury	3	11	704	2 [1, 3]	< 0.001
Pulmonary embolism	4	14	828	2 [0, 4]	0.02
Arrythmia	6	20	828	2 [0, 4]	< 0.001
Empyema	11	28	1883	1 [0, 2]	< 0.001
Wound infection	5	15	1240	1 [0, 2]	< 0.001
Renal dysfunction	2	6	401	1 [0, 3]	< 0.001
Re-expansion pulmonary edema	5	18	1188	1 [0, 3]	0.01
Respiratory failure	7	13	1863	0 [0, 1]	0.24
Acute respiratory distress syndrome	14	27	2889	0 [0, 1]	0.36
Bleeding	7	13	2148	0 [0, 1]	< 0.001
Tumor recurrence at port	2	4	428	0 [0, 1]	0.17
Myocardial infarction	3	5	1253	0 [0, 1]	< 0.001
Pulmonary edema	3	3	561	0 [0, 2]	0.44

Infectious complications included pneumonia (4% [95% CI: 0, 12]), empyema (1% [95% CI: 0, 2]), and wound infections (1% [95% CI: 0, 2]) (Table 1; Figure S2). Other complications included thromboembolism (3% [95% CI: 2, 5]), pulmonary embolism (2% [95% CI: 0, 4]), arrythmia (2% [95% CI: 0, 4]), hypotension (2% [95% CI: 0, 5]), nausea/vomiting (2% [95% CI: 0, 5]), renal dysfunction (1% [95% CI: 0, 3]), tumor recurrence at port (0% [95% CI: 0, 1]), myocardial infarction (0% [95% CI: 0, 1]), and bleeding (0% [95% CI: 0, 1]) (Table 1; Figure S3).

Complications reported each by a single study included postoperative confusion (2%), cough (1%), syncope (1%), perforated gastric/duodenal ulcer (0.5%), fluid loculation requiring fibrinolytics (2%), bronchopleural fistula (3%), red blood cell transfusion (5%), atelectasis (1%), cellulitis (15%), pleural infections (7%), diarrhea (34%), anorexia (37%), hypoxia (2%), hypoxemia (2%), liver dysfunction (6%), wound dehiscence (2%), and hepatic encephalopathy (2%).

## Discussion

Thoracoscopic talc insufflation for MPEs has been found to be associated with several complications. Whereas the incidences of ARDS and respiratory failure were low, many other adverse effects including pain, fever, pneumothorax and pneumonia had high incidence rates. Emphysema, prolonged drainage, prolonged air leakage, and thromboembolism also had considerably higher prevalence. Among other important complications, respiratory insufficiency, lung injury, re-expansion pulmonary edema, empyema, pulmonary embolism, arrythmia, hypotension, and renal dysfunction were observed at low incidence rates.

MPE is a fluid which accumulates between the visceral (lung covering) and parietal (inner chest wall covering) layers of the pleural cavity. Accumulation of MPE starts when either the resorptive capacity of the pleura exceeds or the absorptive capacity of the pleural lymphatic system is reduced. When accumulates in higher volumes, it compresses lungs due to which the function of pulmonary parenchyma is affected that results in dyspnea [41]. Talc, the hydrated magnesium silicate (H_2_Mg_3_(SiO_3_)_4_), is in use for pleurodesis since 1930s. Talc like other agents produces aseptic pleuritis to control accumulation of effusions [41]. Graded talc (talc after removal of small particle size because of their association with adverse events) is more useful than mixed talc.

Approximately 5 g (range 2 to 10), on average, of talc was used by the included studies of this meta-analysis. High doses of talc and small particle-sized talc are found hazardous for pleurodesis. Small particle-sized talc can be systemically absorbed through parietal lymphatics and can induce systemic inflammation [42]. Incidence of hypoxemia and ARDS after talc pleurodesis are thought to be due to lung and systemic inflammation as such events are noted more with the use of mixed talc in comparison with graded talc [43].

In a prospective study in which patients with MPEs were treated with small particle-sized talc, significantly higher levels of proinflammatory cytokines were found in the pleural fluid and serum. Moreover, pleural tumor burden of talc had a positive correlation with proinflammatory cytokines in serum which suggested that advanced stage of tumor induced a stronger systemic reaction to talc [44]. Animal studies with small and large sized talc particles have also shown similar inflammatory responses to talc administration [45].

ARDS is considered as a clinically important adverse effect of thoracoscopic talc pleurodesis as the incidence of ARDS leading to mortality is reported by several studies [24, 46–48]. We have found a low incidence of ARDS in a meta-analysis of 14 studies. Beyond the present study, a 9% incidence of ARDS was observed in a retrospective study of cancer patients with MPEs who were treated with talc pleurodesis. However, a vast majority of patients were treated with talc slurry in this study [46]. In another retrospective study in which patients were treated with talc slurry, the ARDS was observed in 1 of 33 patients [47]. Moreover, a study in which 27 patients were treated with either talc insufflation or talc slurry found no incidence of ARDS in talc insufflation group but 4% in talc slurry group [49]. It is suggested that the risk of acute respiratory complications should not be underestimated and a close monitoring of up to 72 h post-pleurodesis should be considered [21, 24].

It is thought that complications like pain, fever, and pneumothorax may arise from thoracoscopy rather than pleurodesis [22]. However, a prospective study found that fever developed in patients who underwent diagnostic thoracoscopy was mild but those who underwent thoracoscopic talc insufflation had significant fever due to inflammatory reaction [25]. Complications like re-expansion pulmonary edema, which is thought to develop because of the prolonged lung collapse followed by its rapid re-expansion, may require mechanical ventilation, if severe [21]. Whereas the talc pleurodesis has been found to provide relief from dyspnea in majority of the patients [50], dyspnea can be a serious complication after pleurodesis failure or can develop because of the re-accumulation of fluid [18], and in its severe forms it can lead to ARDS [51]. Air leakage can occur upon lung or visceral pleural biopsies or due to the rupturing of tumor nodules during lung re-expansion and is usually managed by progressive suction methods [17].

In a survey of over 800 pulmonologists from 8 countries, it was found that their satisfaction with pleurodesis agents was modest although they preferred talc but reported higher incidence of complications especially respiratory failure with the use of talc in pleurodesis [52]. In another survey of about 200 pulmonologists, respondents reported that pain, fever, empyema, and leukocytosis are common side effects with talc pleurodesis [53]. Retrospective studies are usually considered to have a tendency towards under-estimating complications [25]. In the present meta-analysis, approximately 65% of the included studies were retrospective in design. Thus, such a bias could had impacted the overall outcomes. However, inclusion of a good number of studies can minimize the impacts of such biases and therefore our prevalence estimates for ARDS, pain, empyema, fever, bleeding, arrythmia, pneumonia, wound infections, re-expansion pulmonary edema, emphysema, prolonged air leakage, and pulmonary embolism may have better reliability than other complications.

## Conclusions

Pain and fever were the most frequent and ARDS was among the less observed complications of thoracoscopic talc insufflation. Pneumothorax, and pneumonia were also among the highly prevalent complications. Emphysema, prolonged drainage, prolonged air leakage, and thromboembolism had moderate incidence rates. Among other important complications, respiratory failure, respiratory insufficiency, lung injury, re-expansion pulmonary edema, empyema, pulmonary embolism, and arrythmia were observed at relatively lower incidence rates.

## Supplementary Information

**Additional file 1: Table S1.** Important characteristics of the included studies. **Tables S2a.** Quality assessment with New Castle-Ottawa Scale for the Quality Assessment of the Observational Studies. **Tables S2b.** Quality assessment with Cochrane Risk of Bias Assessment Tool for Randomized Controlled Trials. **Figure S1.** A forest graph showing the pooled mortality rates after thoracoscopic insufflation with talc. **Figure S2.** A forest graph showing the incidence rates of pneumonia, empyema, and wound infections after thoracoscopic talc insufflation. **Figure S3.** A forest graph showing the incidence rates of persistent pain, fever, thromboembolism, pulmonary embolism, arrhythmia, hypotension, nausea/vomiting, renal dysfunction, tumor recurrence at port, and myocardial infarction after thoracoscopic talc insufflation.

## Data Availability

All data generated or analyzed during this study are included in this published article.

## Abbreviations

MPEs: Malignant pleural effusions
CI: Confidence interval
