# Supplementary material for: Complications of thoracoscopic talc insufflation for the treatment of malignant pleural effusions: a meta-analysis
Source: J Cardiothorac Surg. 2021 May 4;16:125. doi: 10.1186/s13019-021-01475-1 (PMC8097876; doi:10.1186/s13019-021-01475-1)
Supplement: Supplementary file 1 — Additional file 1: Table S1. Important characteristics of the included studies. Tables S2a. Quality assessment with New Castle-Ottawa Scale for the Quality Assessment of the Observational Studies. Tables S2b. Quality assessment with Cochrane Risk of Bias Assessment Tool for Randomized Controlled Trials. Figure S1. A forest graph showing the pooled mortality rates after thoracoscopic insufflation with talc. Figure S2. A forest graph showing the incidence rates of pneumonia, empyema, and wound infections after thoracoscopic talc insufflation. Figure S3. A forest graph showing the incidence rates of persistent pain, fever, thromboembolism, pulmonary embolism, arrhythmia, hypotension, nausea/vomiting, renal dysfunction, tumor recurrence at port, and myocardial infarction after thoracoscopic talc insufflation. [file 13019_2021_1475_MOESM1_ESM.docx]

| Table S1: Important characteristics of the included studies | | | | | | | | | | |
| --- | --- | --- | --- | --- | --- | --- | --- | --- | --- | --- |
| Study | **n** | **Design** | **Follow-up (months)** | **Talc dose (grams)** | **Age (years)** | **% females** | **Chest tube drainage (days)** | **Hospital stay (days)** | **Success rate (%)** | **Post-pleurodesis survival (mo)** |
| Aeloney 2005 | 26 | RET | 24±16 | 2.5-5 | 68±8.3 | 12 | 2.8±1.4 | 3.9±2.7 |  | 23.8±16.3 |
| Arapis 2006 | 273 | RET | 8±11 | 5 | 60.6±13.2 | 64 | 3.6±3.2 | 7.1±3.2 | 78.8 | 9.2 |
| Barbetakis 2010 | 400 | RET | 40±10 | 6 | 63.2±9.5 | 35 | 6±1 | 7±1 | 85 | 7.3 |
| Bhatnagar 2020 | 166 | RCT | 6 | 4 | 68±11 | 58 |  | 12.1±13 | 90 |  |
| Cardillo 2012 | 611 | RET | 64 | 5 | 61.1±10 | 39 |  | 5.8±4.5 | 92.7 |  |
| Daniel 1990 | 20 | RET |  | 5 | 1–78 |  |  |  | 90 |  |
| de Campos 2001 | 501 | RET | 1–86 | 2 | 54.5±15.8 | 59 | 6.15±5 |  | 95.4 |  |
| Debeljak 2006 | 22 | RET |  | 3-5 | 62.2±12.2 |  | 4.8±3.5 |  | 77 |  |
| Diacon 2000 | 17 | RCT |  | 5 | 65.3±2.8 | 59 | 4.7±0.5 |  |  | 4.4 |
| Dresler 2005 | 242 | RCT | 6 | 4-5 | 62.5±11.7 | 53 |  |  | 73 |  |
| Froudarakis 2006 | 18 | RET | 3 | 4 | 63.1±15.5 | 33 | 3.4±1.4 |  | 94.4 |  |
| Fysh 2013 | 87 | RET |  |  | 70±10.4 | 7 |  | 6±1.3 | 69 | 7.7 |
| Gonzalez 2010 | 138 | RET |  | 6 | 67±13 | 47 | 4.3±3.3 | 5.5±4 |  | 9.5 |
| Guo 2012 | 128 | PROSP | 12 | 2-4 | 61.6±10.8 | 47 | 4.03±0.5 |  | 72.8 |  |
| Hunt 2012 | 50 | RET |  | 4 | 66 | 54 |  | 8 |  |  |
| Inoue 2013 | 57 | PROSP |  | 4 | 68.1±10.5 | 68 | 4.4±2.2 |  | 90.6 |  |
| Janssen 2007 | 558 | PROSP |  | 4 | 64.4±11 | 51 |  |  |  |  |
| Kolschmann 2005 | 102 | RET-CS | 12 | 8 | 66±11 | 44 | 6±4.1 |  | 89 |  |
| Laisaar 2006 | 98 | RET |  | 5 | 59.6±10 | 71 | 3±3.2 | 7±10.5 |  | 8.4 |
| Leuzzi 2011 | 401 | RET |  | 4 |  |  |  |  |  |  |
| Love 2003 | 55 | RET |  | 4-5 | 68.1±9.3 | 62 |  |  |  |  |
| Mohsen 2011 | 22 | RCT | 23±10 | 4 | 48.2±9.9 |  |  |  | 91 | 7.3±24 |
| Reddy 2011 | 30 | RET | 6 | 5 | 65.2 | 67 | 16.7±31 | 3.1±3 | 92 |  |
| Stefani 2006 | 72 | PROSP | 9±10 | 6 | 64 | 53 |  |  | 88 | 11.2 |
| Viallat 1996 | 360 | RET | 12 | 4.5 | 60±1 |  | 5.3±0.2 |  | 85.4 | 6.4 |
| Yim 1996 | 28 | RCT | 10 | 5 | 60±9.4 | 64 | 3.8±2.4 | 7.6±2.8 |  |  |
| Abbreviations: PROSP, prospective; RCT, randomized controlled trial; RET, retrospective; OL, open-label; CS, case series | | | | | | | | | | |

| Tables S2a: Quality assessment with New Castle-Ottawa Scale for the Quality Assessment of the Observational Studies | | | | | | | | |
| --- | --- | --- | --- | --- | --- | --- | --- | --- |
| Study | **Representativeness of exposed cohort** | **Selection of non-exposed cohort** | **Ascertainment of exposure** | **Demonstration that outcome of interest was not present at start of study** | **Comparability of cohorts on the basis of the design or analysis** | **Assessment of outcome** | **Was follow-up long enough for outcomes to occur** | **Adequacy of follow up completion of cohorts** |
| Aeloney 2005 | * |  | * | * |  | * | * | * |
| Arapis 2006 | * |  | * | * |  | * | * | * |
| Barbetakis 2010 | * |  | * | * | * | * | * | * |
| Cardillo 2012 | * |  | * | * |  | * | * | * |
| Daniel 1990 | * |  | * | * |  | * |  |  |
| de Campos 2001 | * |  | * | * |  | * | * | * |
| Debeljak 2006 | * |  | * | * |  | * |  |  |
| Froudarakis 2006 | * |  | * | * | * | * | * | * |
| Fysh 2013 | * |  | * | * | * | * |  |  |
| Gonzalez 2010 | * |  | * | * |  | * | * | * |
| Guo 2012 | * |  | * | * | * | * | * | * |
| Hunt 2012 | * |  | * | * | * | * |  |  |
| Inoue 2013 | * |  | * | * |  | * | * | * |
| Janssen 2007 | * |  | * | * |  | * |  |  |
| Kolschmann 2005 | * |  | * | * | * | * | * | * |
| Laisaar 2006 | * |  | * | * | * | * | * | * |
| Leuzzi 2011 | * |  | * | * |  | * |  |  |
| Love 2003 | * |  | * | * | * | * | * | * |
| Reddy 2011 | * |  | * | * |  | * |  |  |
| Stefani 2006 | * |  | * | * | * | * | * | * |
| Viallat 1996 | * |  | * | * |  | * | * | * |
| Asterisk represent fulfillment of the criterion. | | | | | | | | |

| Tables S2b: Quality assessment with Cochrane Risk of Bias Assessment Tool for Randomized Controlled Trials | | | | | | | | |
| --- | --- | --- | --- | --- | --- | --- | --- | --- |
| Study | **Other bias** | **Selective reporting** | **Incomplete outcome data** | **Blinding of outcome assessment** | **Blinding of participants /personnel** | **Allocation concealment** | **Random sequence generator** | |
| Bhatnagar 2020 | L | L | L | L | H | H | L | |
| Diacon 2000 | L | L | L | H | U | H | L | |
| Dresler 2005 | L | L | L | U | U | H | L | |
| Mohsen 2011 | L | L | L | U | U | H | L | |
| Yim 1996 | L | L | L | U | U | H | L | |
| Legends: H: high risk; L: low risk; M: mediocre risk U: unclear risk | | | | | | | |  |


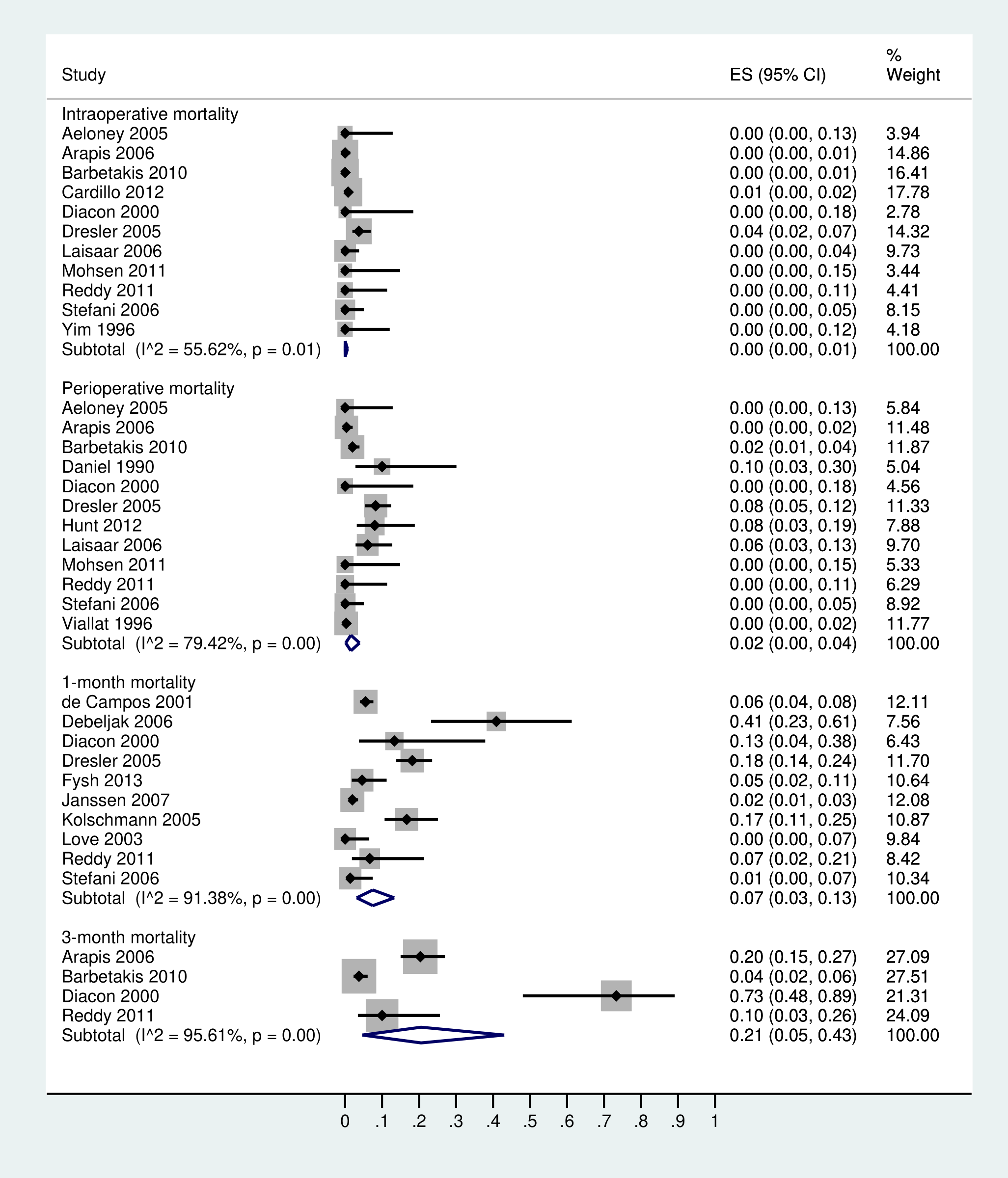


Figure S1: A forest graph showing the pooled mortality rates after thoracoscopic insufflation with talc.


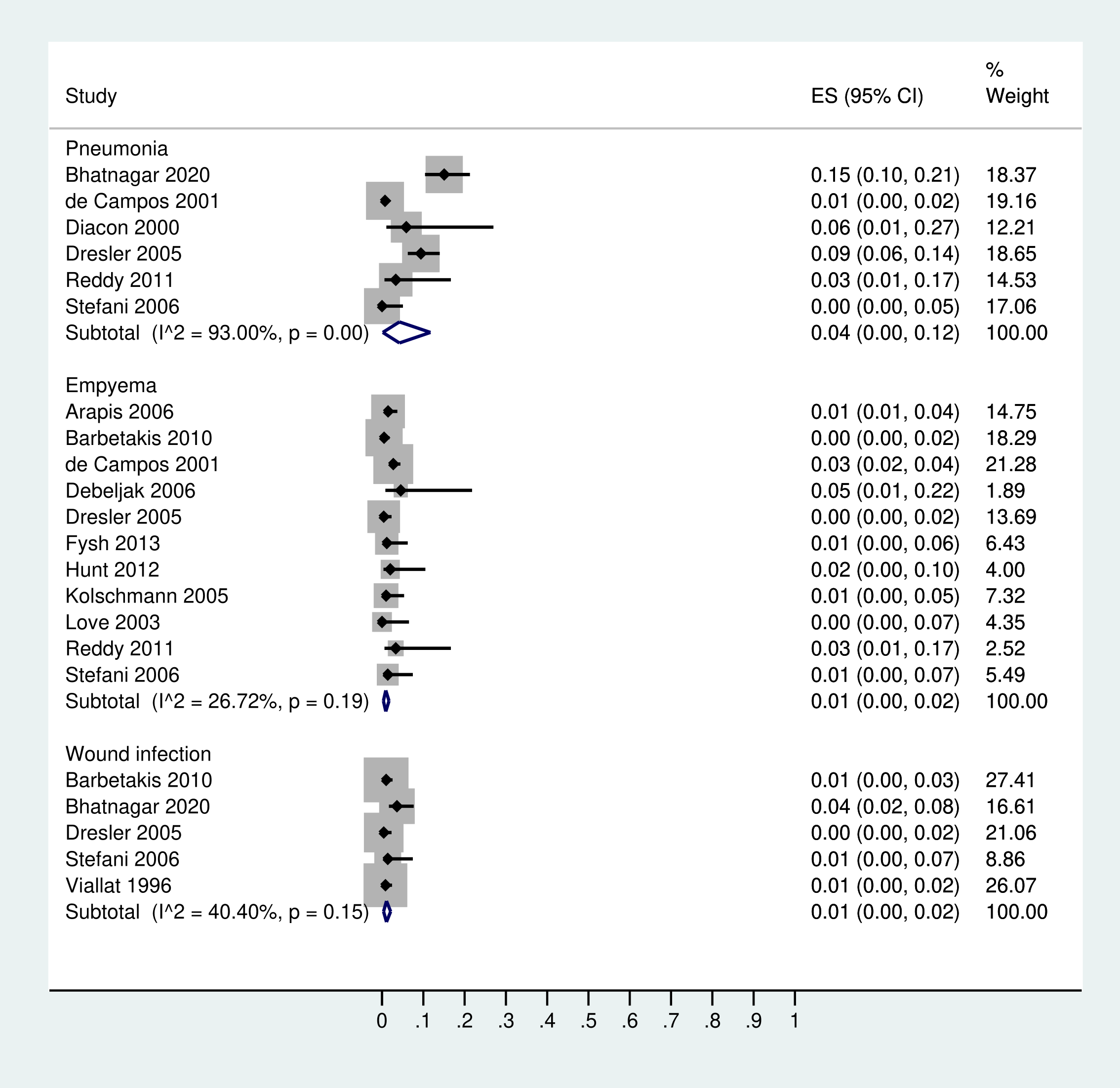


Figure S2: A forest graph showing the incidence rates of pneumonia, empyema, and wound infections after thoracoscopic talc insufflation.


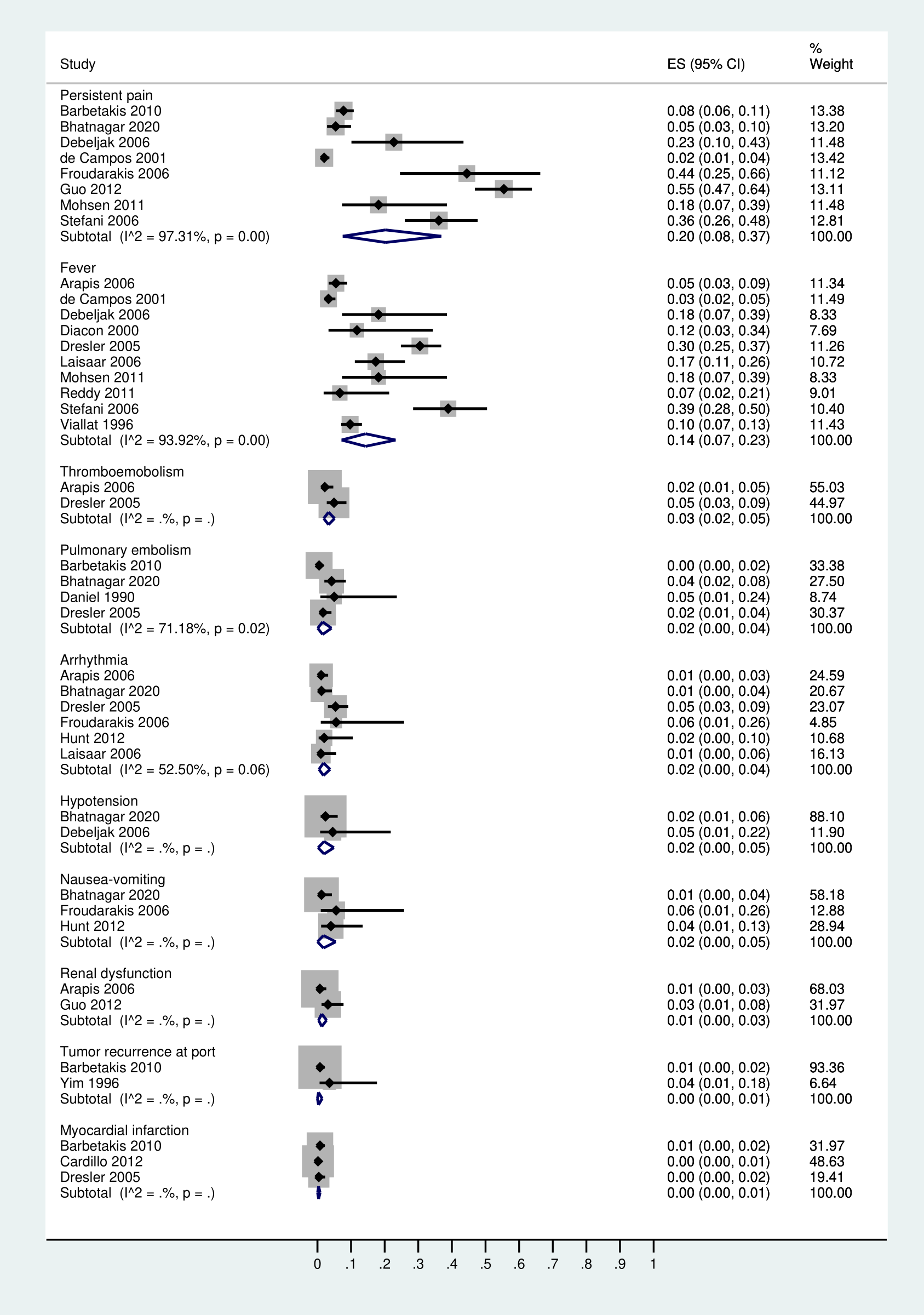


Figure S2: A forest graph showing the incidence rates of persistent pain, fever, thromboembolism, pulmonary embolism, arrhythmia, hypotension, nausea/vomiting, renal dysfunction, tumor recurrence at port, and myocardial infarction after thoracoscopic talc insufflation.
